# Assessing the Impact of Human Activities on British Columbia’s Estuaries

**DOI:** 10.1371/journal.pone.0099578

**Published:** 2014-06-17

**Authors:** Carolyn K. Robb

**Affiliations:** School of Environment and Management, Royal Roads University, Victoria, British Columbia, Canada; University of Auckland, New Zealand

## Abstract

The world’s marine and coastal ecosystems are under threat and single-sector management efforts have failed to address those threats. Scientific consensus suggests that management should evolve to focus on ecosystems and their human, ecological, and physical components. Estuaries are recognized globally as one of the world’s most productive and most threatened ecosystems and many estuarine areas in British Columbia (BC) have been lost or degraded. To help prioritize activities and areas for regional management efforts, spatial information on human activities that adversely affect BC’s estuaries was compiled. Using statistical analyses, estuaries were assigned to groups facing related threats that could benefit from similar management. The results show that estuaries in the most populated marine ecosections have the highest biological importance but also the highest impacts and the lowest levels of protection. This research is timely, as it will inform ongoing marine planning, land acquisition, and stewardship efforts in BC.

## Introduction

Estuaries are one of the world’s most biologically productive ecosystems [1], [2], [3], providing ecosystem services valued as high as $22,000 US per hectare per year [4]. In British Columbia, Canada (BC), calculations have shown that estuarine habitats sequester a minimum of 180,000 tonnes of carbon annually [5] and Kelsey [6] estimated that 80% of all coastal wildlife use estuaries. However, anthropogenic threats have made estuaries one of the most degraded ecosystems on earth [3], [7] and these threats are expected to increase in prevalence and severity as coastal populations continue to expand [7].

Many estuarine threats originate within the watersheds that drain through the estuaries [7], [8]. The conversion of land for agriculture, forestry, or residential and industrial development causes habitat loss or fragmentation [7], [9]. These changes in land use can trigger an increase in sediment, debris, nutrient, and pollutant levels in estuaries that smothers or entangles organisms or causes eutrophication, hypoxia, and anoxia [1], [3], [7], [10], [11]. Development that expands impervious surfaces within the watershed can increase water flow into estuaries, particularly during storm events [7], while dams impede water inputs [1], [7], [9]. As the climate warms, increased precipitation and more frequent storm surges are also anticipated in BC [9]. All of these hydrological changes can disrupt water filtration [7] and alter estuarine productivity and trophic structure [12], [13].

Other threats originate in the marine environment. Development and industrial activities within estuaries, such as log-handling, aquaculture, dredging, and vessel traffic, can result in habitat loss or degradation, environmental contamination, shoreline erosion, the re-suspension of sequestered carbon, and the invasion of alien species [1], [7], [9], [10], [12]. Commercial and recreational fishing can alter the composition of estuarine species through the preferential removal of target species [7], [12]. Shoreline armouring to stabilize and protect coastal developments limits the ability of estuaries to retreat inland as the climate warms and sea levels rise and may therefore cause habitat and shorebird losses [1], [9], [13], [14]. Higher sea temperatures may increase the severity of eutrophication in estuarine salt marshes [15]. Lastly, the acidification of estuarine waters may inhibit the growth of shelled organisms [16] and increase the bioavailability of trace metals from pollution [17].

The continuing degradation of estuarine habitats has led to a variety of research efforts. In BC, the Pacific Estuary Conservation Program (PECP), a partnership of government and non-government organizations, has mapped the boundaries of 442 estuaries and designed regional scale projects to prioritize estuaries for conservation based on their biological importance [2]. Further research has found that 38% of BC estuaries had economic tenures within the intertidal area [18]. Merrifield *et al.* [1] recently completed a regional assessment of threats to estuaries along the west coast of the United States which face similar threats to BC estuaries [19]. The researchers found that only 16% of surveyed estuaries faced few or no anthropogenic threats while 25% of estuaries had moderate levels of all assessed threats [1]. However, a systematic investigation of human activities affecting BC estuaries has not yet been completed.

In BC, the management of estuarine areas and activities requires the coordination of several government agencies. Subtidal and intertidal areas are typically public land while upland areas can be publicly or privately owned. The BC government regulates the use of public lands by issuing tenures but the ongoing activities of some industries with tenures, such as aquaculture, are managed federally. Federal jurisdiction also pertains to all organisms and most activities within the water column, areas designated for port facilities, and activities on or beneath the seabed in offshore areas [20]. In public areas, the BC government and several federal agencies have established protected areas that, to varying degrees, shield estuarine habitat from human activities [21]. In private areas, the PECP works to secure and rehabilitate important estuarine habitats through direct land purchases [2], [22].

Recent reports attribute estuary degradation to a failure of management efforts that have focused predominantly on individual industrial sectors [23], [24], [25] without examining the cumulative impacts of activities [24], [26], [27], [28] or the ecosystem services on which the activities rely [29]. There is a growing consensus that a broader management focus, termed ecosystem-based management (EBM), is necessary to tackle the problems facing marine and coastal environments [23], [24], [26], [27], [30], [31], [32]. EBM is place-based and considers both the spatial variations of, and the interactions between, the ecological, physical, and human elements of ecosystems [24], [31]. In Canada, Fisheries and Oceans Canada (DFO) has developed a framework for estuarine, coastal, and marine management following the principles of EBM [23].

To implement EBM effectively, ecosystem elements must be mapped and the impacts of human activities assessed [23], [24], [28], [33]. This research uses geographic information systems (GIS) to investigate the spatial distribution of human activities that affect BC estuaries, incorporating threats relevant to BC that have not been considered previously. Cluster analysis is used to categorize estuaries based on the presence and magnitude of threat variables. Unlike in previous research [1], the spatial range of influence of each threat is incorporated to generate a more realistic spatial representation of each threat [33], the cluster analysis uses a non-hierarchical approach to allow the use of non-normal variables, and results are compared to assessments of each estuary’s biological importance and conservation status. By identifying estuaries facing similar threats, this work gives context to previous prioritization efforts that focused solely on ecological factors [2], informs regional planning efforts, and helps prioritize limited conservation and research resources to ensure vital ecosystem services are sustained.

## Methodology

### Study Area

**Figure 1:**
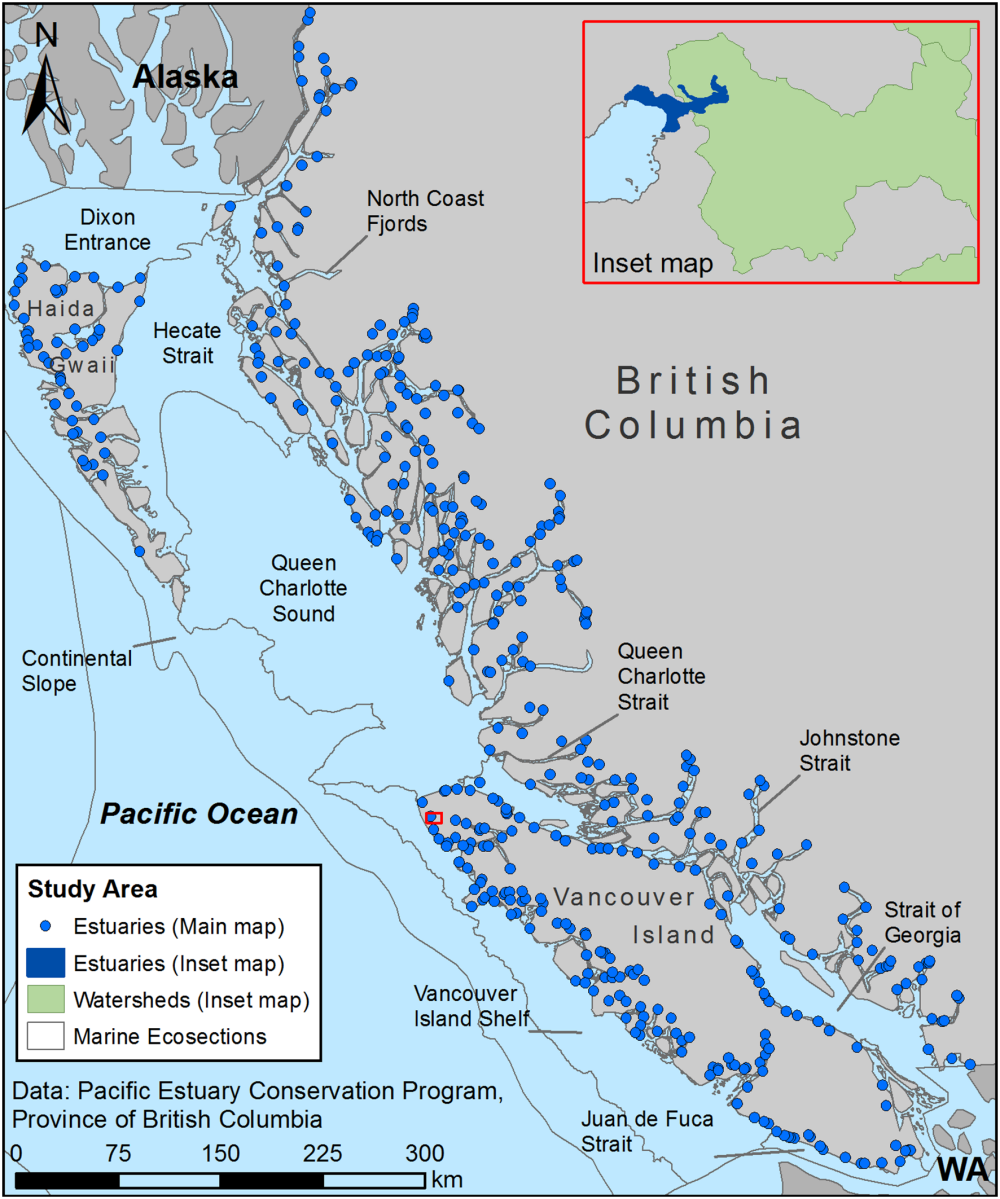
Study area. Description: This research investigates the presence of threats within BC estuaries, which are found along the BC coastline from the southern border with Washington to the northern border with Alaska. The inset map shows a typical estuary and watershed.

BC’s estuaries, comprising less than three percent of the coastline, form the study area for this research (Figure 1). I used estuaries and associated watersheds as the basic spatial units for the analysis, referred to as “estuary-watershed systems”, because they are considered the complete geographic boundaries for estuarine management [8], [34].

### Estuary and Watershed Data Preparation

In a desktop GIS, I overlaid spatial data for watersheds and estuaries (Table S1) to identify the watershed associated with each estuary (Figure 1). I confirmed that overlapping estuaries and watersheds shared the same primary waterway using attribute information found in both datasets. Estuaries that could not be linked to a unique watershed were removed. I added attribute information on importance for waterbirds [2] and carbon sequestration [5] to each estuary based on estuary name. I also overlaid the estuary-watershed systems with spatial data on legislated protected areas and privately conserved lands (Table S1) to determine the count and total area of conservation designations within each system. Lastly, I overlaid the systems with BC’s marine ecosections, which are planning areas with similar physical and biological characteristics, to determine the average estuarine importance and conservation values by ecosection (Table 1).

**Table 1:** Average conservation importance, protection level, and threat status of BC estuaries, grouped by marine ecosection.

Marine Ecosection	Importance for Waterbirds	Count ofThreats	Count ofProtectedAreas	Proportion ofAreaProtected (%)	AverageWatershedArea (ha)	Average EstuarineArea (ha)
Continental Slope	3.4	6.2	1.6	57.9%	4,328.3	17.0
Dixon Entrance	3.2	6.1	1.2	42.4%	6,646.5	140.0
Hecate Strait	3.5	7.0	1.3	22.4%	3,859.1	55.7
Johnstone Strait	3.6	8.9	0.8	12.8%	5,724.1	64.0
Juan de Fuca Strait	3.7	9.1	2.3	11.5%	5,019.6	25.1
North Coast Fjords	3.6	6.6	1.3	27.8%	6,514.0	192.7
Queen Charlotte Sound	3.5	5.2	1.3	20.3%	4,101.3	29.4
Queen Charlotte Strait	3.3	9.3	0.9	3.1%	5,542.8	125.4
Strait of Georgia	3.2	11.0	2.4	4.2%	7,132.2	783.3
Vancouver Island Shelf	3.4	8.9	1.0	13.3%	5,255.5	45.9

### Threat Data Preparation

To identify the primary anthropogenic threats facing BC estuaries, I reviewed the relevant literature, focusing on research on estuaries and estuarine habitats, such as salt marshes, seagrass beds, and sand flats, from BC and the Pacific coast of North America (Table 2). Spatial data for the primary threats (Table S2) were chosen based on their relevance to estuaries, spatial coverage, quality, resolution, and currency and used to create a comprehensive spatial layer for each threat along the BC coast. While gaps did exist (Table S3), data were available for the majority of the identified threats.

**Table 2:** The incidence of estuarine threats within the literature relevant to BC estuaries and estuarine habitats, such as salt marshes, seagrass beds, and sand flats.

	Key sources used to identify threats to estuaries and estuarine habitats						
Estuarine threats	[9]	[8]	[19]	[7]	[1]	[2]	[35]
Terrestrial							
Development in watershed	X	X	X	X	X	X	
Agriculture in watershed	X	X	X	X	X		
Forestry in watershed		X	X	X	X		
Watershed pollution	X	X	X	X	X	X	X
Freshwater diversions	X	X	X	X	X	X	X
Shoreline armouring in estuary	X	X	X	X	X		X
Impaired waterways		X	X	X	X	X	
Debris/litter				X		X	X
Marine							
Development of estuary shoreline	X	X	X	X	X	X	X
Port facilities in estuary	X	X		X	X		X
Dredging	X	X	X	X			X
Marine pollution				X	X	X	X
Aquaculture	X		X	X	X		X
Resource extraction				X		X	X
Introduced species	X	X	X	X		X	X
Recreation		X		X			
Increased atmospheric carbon dioxide							
Sea level rise	X	X		X	X	X	X
Sea surface temperature		X					X
Ocean acidification							X
Change in future precipitation	X	X			X		
Ecosystem focus (location)	Est* (BC)	SM (WC US)	Est (WC NA)	Est (global)	Est (WC US)	Est (BC)	SM (CA)

*Abbreviations: Est = estuary; SM = salt marsh; BC = British Columbia; WC = west coast; US = United States of America; NA = North America; CA = California.

Several spatial analysis methodologies were used to compile the data (Table S2) for further statistical analysis. I classified land use data into agricultural or urban areas, combining the urban areas with railroads and road networks. I identified armoured shorelines by combining dikes and manmade shoreline segments. I selected the locations of freshwater diversions from a point dataset of obstructions. To identify areas that had been clearcut over the past decade, I extracted polygons with a stand age of 10 or less from a provincial dataset of harvest depletion. I located sources of point and nonpoint watershed pollution from data on pulp mills, mines, and areas closed due to contamination. Lastly, I calculated the percent change in modeled future precipitation using data on current (1961–1990 normal) and future (2080’s CGSM A2x scenario) precipitation from ClimateBC [36] and the methodology employed by Merrifield *et al.* [1].

The compilation of marine threat data (Table S2) was guided by the best available data identified by the BC Marine Conservation Analysis (BCMCA) [37]. I obtained provincial tenure data to identify public areas leased to marine-related industrial activities and confirmed site usage with site-specific finfish aquaculture production data from DFO or by visually confirming the presence of infrastructure, such as aquaculture pens or log storage sites, using Google Earth 6.1 [38] satellite imagery. I merged the tenures to create features for shoreline development and aquaculture. I transformed the tenures for marinas, commercial wharves, and ferry terminals into points and combined them with BCMCA datasets representing marinas, anchorages, and tow boat reserves. I obtained the point locations of dredging operations and extracted data for large vessels from a gridded dataset of vessel traffic (Table S2). Smaller vessels were not well represented in this dataset so I used linear data on recreational boating routes to represent the distribution of small vessels. I combined fisheries datasets to create features for commercial and recreational fisheries. To retain the catch values available in the commercial fishing datasets, the values for all fisheries were converted to kg and summed. No high resolution datasets were available for projected ocean acidification, sea surface temperature, or sea level rise within BC’s coastal zone (Table S3).

### Threats within Estuaries

The maximum spatial range of influence of each activity was determined from zone values specified by Ban *et al.* [33] and Quigley and Hall [39] (Table S2). Incorporating zones more realistically represents the full spatial extent of each threat [33] and ensures that all relevant threats are considered, though the spatial footprint of associated infrastructure may not overlap directly with an estuary or watershed. I buffered each threat by the zone value to create polygons representing the larger zone of influence. Spatial ranges were not calculated for features with low data resolution [33], features where the zone of impact was not known, or features that already incorporated the zone of impact (Table S2). The spatial range was calculated for large vessel traffic, despite the low data resolution, due to the wide influence of this feature and because the original data had been fit to a coastline that did not incorporate the mapped estuaries in all areas.

To determine the impact of the threats within each estuary-watershed system, I overlaid the buffered threats with the estuaries and watersheds. To standardize for variation in the size of the systems, I calculated the proportion of each system affected by the activity. For point features, I determined the density of points within each estuary-watershed system. For a few gridded features, information on level of use was available and used to better represent their impact spatially. Because the impact of vessel traffic, such as ship wake, can extend into an estuary from outside, I spatially joined the vessel traffic data with the estuaries and summed the number of vessel hours for all overlapping grid cells to calculate the total impact. For commercial fishing, I intersected the grid of fisheries catch data (Table S2) with the estuary polygons and summed the total catch of the overlapping grid cells proportional to the area of overlap to estimate the catch taken within each estuary. I calculated the average precipitation change for each estuary-watershed system using zonal statistics, which examined and combined the values of cells in the gridded precipitation dataset that fell within the estuary-watershed systems.

I counted the number of threats affecting each estuary and determined the proportion of the estuary and watershed area covered by those activities. The average values were compared among estuaries in different marine ecosections (Table 1) and estuaries with different conservation status and biological importance.

### Statistical Analyses

I ran cluster analyses using Systat 13 [40] to group the estuaries based on the presence and magnitude of the threats (Table 3). Pairwise correlations between threats were examined to ensure the included threats were independent and considered equal in the analysis. Most of the threat variables had negatively skewed distributions and were log-transformed to minimize the impact of a few scattered higher values [28]. To determine the optimal number of clusters, I performed preliminary analyses with hierarchical clustering and the Ward method. I used non-hierarchical K-medians for the final cluster analysis because the method is resistant to non-normal variables and outliers [41]. Euclidean distance was used to determine the distance of each site from the centers of each cluster.

**Table 3:** Average values of the threats affecting BC estuaries, grouped into the three clusters generated by the K-medians cluster analysis.

Cluster	Urban (%)	Tenures (%)	Shoreline Armouring (m/ha)	Aquaculture (%)	Ports (%)	Dredging (%)	WatershedPollution (%)	Agriculture (%)
1	67.8%	5.4%	5.7	1.7%	61.8%	0.6%	12.6%	0.2%
2	92.1%	7.8%	9.0	11.4%	21.4%	0.5%	32.0%	0.4%
3	100.0%	17.8%	38.2	18.6%	72.4%	7.7%	87.8%	14.5%
**Cluster**	**Freshwater** **Diversion (count/ha)**	**Forestry (%)**	**Contamination** **Closures (%)**	**Commercial** **Fishing** **(catch in kg)**	**Recreational** **Fishing (%)**	**Large Vessel** **Traffic** **(hours)**	**Small Vessel** **Traffic** **(%)**	**Future Precipitation (% change)**
1	0.000010	15.4%	3.7%	890.8	19.2%	605.1	75.4%	19.2
2	0.000028	70.3%	3.1%	1,697.9	58.4%	3,650.7	67.5%	16.9
3	0.000075	52.8%	66.3%	23,345.3	37.8%	19,439.6	81.9%	15.8

I mapped the results and generated spatial statistics for the clusters, including the average number of threats and the proportion of each estuary affected by each threat (Tables 3 and 4). I also analyzed the distribution of the clusters among ecosections, sites with a conservation designation, and estuaries considered biologically important (Table 4).

**Table 4:** Average conservation importance, protection level, and threat status of BC estuaries, grouped into the three clusters generated by the K-medians cluster analysis.

Cluster	Number of Estuaries	Importance for Waterbirds	Priority Sites for Carbon Sequestration	Count of Threats	Count of Protected Areas	Proportion of Area Protected (%)	Average Estuarine Area (ha)	Average Watershed Area (ha)
1	180	3.6	0	6.6	1.3	34.4%	88.7	5,918.8
2	127	3.5	2	8.0	0.9	9.5%	163.6	6,034.4
3	69	3.1	6	11.1	2.1	6.7%	521.8	6,129.8

## Results

### Importance and Conservation Status of Estuaries

The average importance of estuaries for waterbirds was similar among all ecosections but priority carbon sequestration sites were only found within the Strait of Georgia and North Coast Fjords ecosections. Federally and provincially legislated protected areas overlapped 58.5% of estuary-watershed systems, predominantly in the North Coast Fjords ecosection. On average, 21% of estuarine and watershed area was covered by a legislated protected area, with the highest proportions in north coast ecosections. A further 27 estuary-watershed systems, primarily on the south coast, overlapped a privately conserved area, with an average of 1.4% of the estuarine and watershed area protected. In total, 61% of estuary-watershed systems had at least one private or legislated protected area.

### Threats in Estuaries

**Figure 2:**
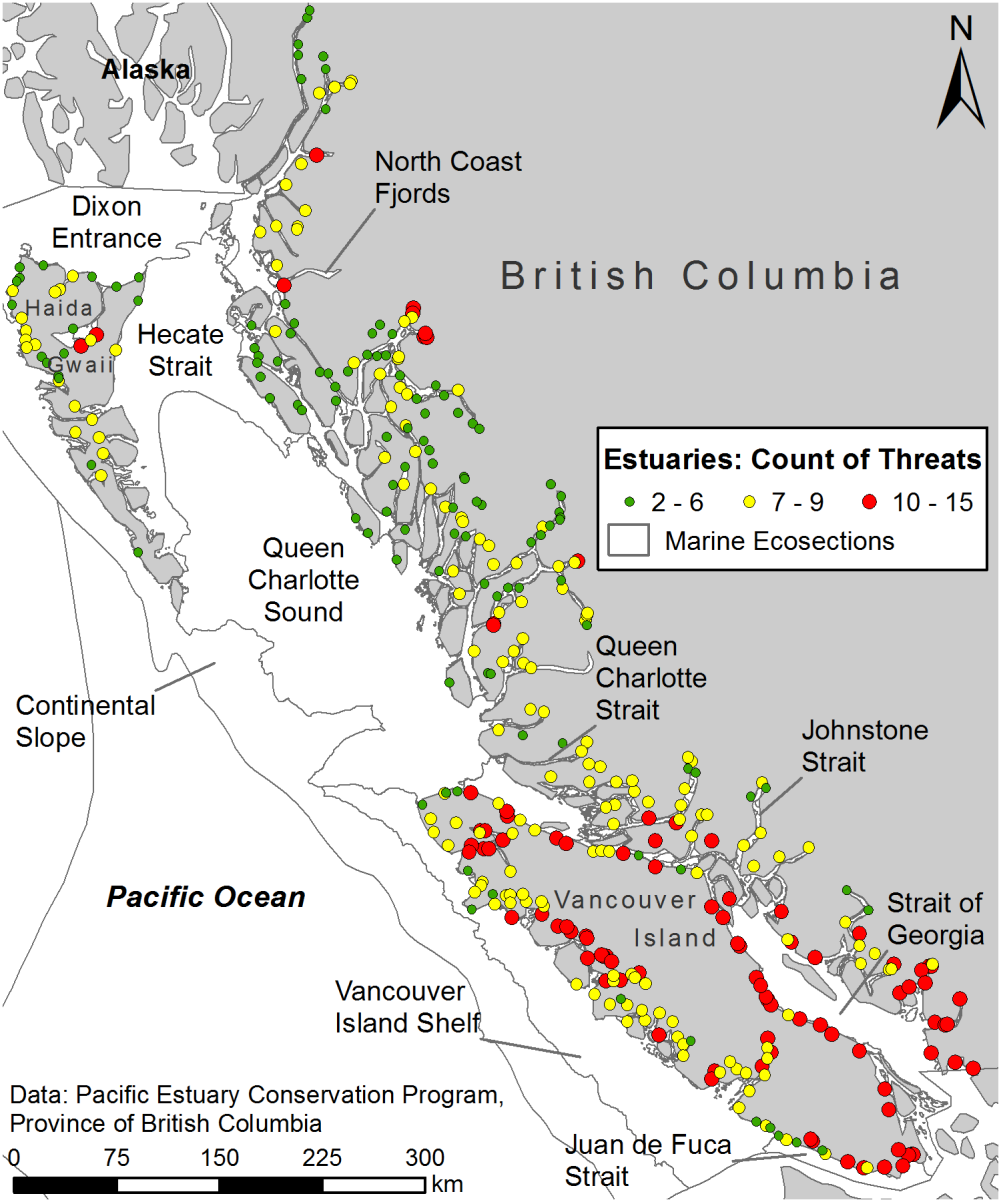
Count of threats by estuary-watershed system. Description: Estuary-watershed systems are shown classified based on the number of the 16 possible threats found within their bounds. The classification uses Jenks natural breaks which maximizes the variance between the classes. The number of threats faced by an estuary is greatest in the more heavily populated south coast ecosections.

Of 442 possible estuaries, 376 were joined to adjacent watersheds and included in the analysis. The literature review uncovered 20 threat categories and, based on the available data, 16 threat variables were created (Table S2). A spatial dataset showing the threat distribution within each estuary is available from the author. Change in future precipitation was the only threat to overlap with all estuary-watershed systems but all systems overlapped at least two threats (Figure 2). No system overlapped all of the threats, though two systems in the Strait of Georgia ecosection were affected by 15 threats. Overall, there was an average of 7.9 threats per estuary-watershed system but systems within the Strait of Georgia ecosection averaged 11.0 threats and four other ecosections, also on the south coast, had a higher than average number of threats per estuary-watershed system (Table 1).

Estuary-watershed systems with the highest importance to waterbirds had the highest counts of threats. Systems with a protected area, either legislated or privately conserved, faced an average of 7.6 threats. Estuary-watershed systems with less area protected faced a greater average number of threats and those facing the fewest threats were the most protected. Systems with a legislated protected area faced fewer average threats than those with a privately conserved area.

### Cluster Analysis

**Figure 3:**
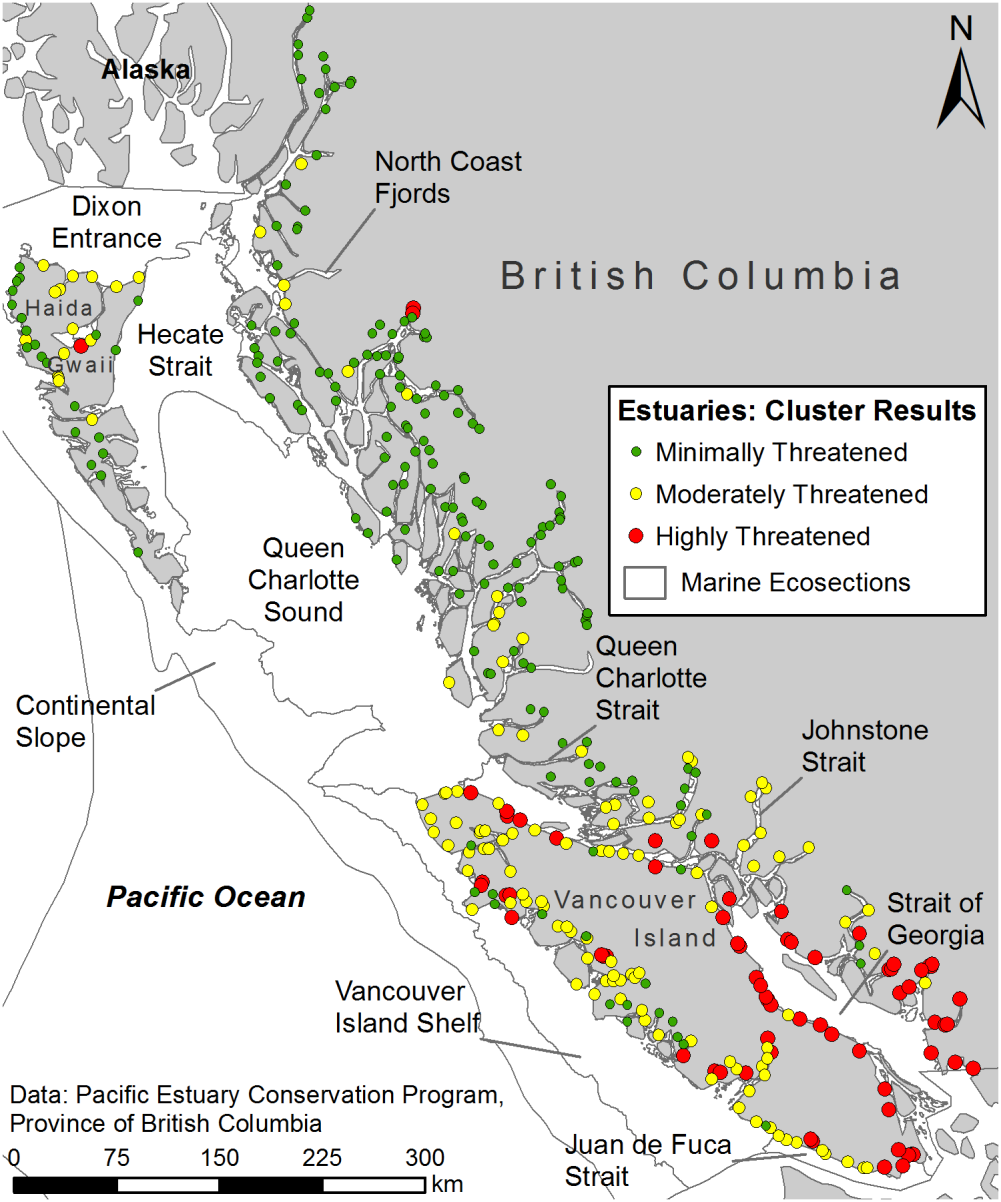
Distribution of K-medians cluster results. Description: Estuary-watershed systems are shown distributed into clusters that resulted from the K-medians cluster analysis based on the presence of 16 threat variables. Similar to the count of threats, the clusters highlight a north-south gradient, with highly threatened estuaries primarily observed in the south coast ecosections and minimally threatened estuaries predominantly observed in the north coast ecosections.

The cluster analysis revealed three optimal clusters (Figure 3). Cluster 3, representing estuaries with the greatest importance for waterbirds and carbon sequestration (Table 4), was the cluster with the greatest impact from most of the threat variables (Table 3). These 69 estuaries were found primarily in the south coast ecosections, dominated by the Strait of Georgia ecosection. Cluster 3 had the greatest average number of protected areas within each estuary, but the smallest average proportion of area protected. Cluster 2 represented 127 estuaries moderately affected by most threat variables and the highest impact from forestry and recreational fishing. The majority of estuaries in this cluster were found on the south coast in the less populated Vancouver Island Shelf ecosection. Cluster 1 faced minimal impacts from most threats but had a higher than average future change in precipitation. Cluster 1 also had the most estuaries, located predominantly within the North Coast Fjords ecosection. Based on average area conserved, these estuaries were also the most protected.

## Discussion

A variety of local approaches have been important for estuary conservation, including the creation of legislated protected areas, the purchase of sites for private conservation, and the establishment of estuary management plans. A conservation designation was found within 61% of the estuary-watershed systems but systems in the north coast ecosections had greater protection than their southern counterparts (Table 1). The biased placement of legislated conservation designations in less threatened areas further from human settlements, and therefore less costly to protect, matches global trends for terrestrial protected areas [42]. Estuary-watershed systems with privately conserved areas faced a greater number of threats than those with legislated protected areas. As Brooks *et al.* [42] note, people most value “what is close to them”. Privately protected areas, often negotiated by the PECP with landowners, are found primarily along the south coast, where the greatest concentration of humans, and industrial activities, is found [10], [17].

In 2002, a review of estuary management plans in BC identified only nine completed plans, predominantly for estuaries on the south coast [43]. Since that time, planning has been completed for two additional estuaries but some of these plans, particularly those with limited stakeholder support, have taken up to 20 years to finalize [43] and some have not been fully implemented (K. Moore, Environment Canada, personal communication). With uneven estuarine protection along the coast and management plans for less than 2% of mapped estuaries, it is apparent that local efforts alone will not be sufficient to conserve estuarine habitats effectively into the future.

To ensure the maintenance of biodiversity, a regional approach based on the principles of EBM offers a more comprehensive, efficient, and cost-effective alternative to site-specific protection and planning [30], [44]. The PECP’s efforts to map and rank estuaries began the work of looking at BC’s estuaries through a regional lens. However, the PECP lacked the comprehensive anthropogenic data necessary to complete the ranking [2]. Identifying clusters of estuaries that face similar threats can highlight priority activities and areas where coordinated management could achieve a higher return on investment.

### Highly Threatened Estuaries

Areas with high diversity or importance and high cumulative impacts are considered conservation priorities to protect species and habitats that would otherwise be lost to habitat modification [28], [42]. Identified as highly important for waterbirds and carbon sequestration, the highly threatened estuaries in Cluster 3 qualify as conservation priorities (Table 4). These estuaries were located predominantly within the heavily populated south coast ecosections (Figure 3), which correlates with results from previous research [18], [33] as well as the simpler count of threats by estuary (Table 1 and Figure 2).

Protected areas that prohibit human activities are a prominent means of conserving estuaries [23] but the systems in Cluster 3 had the lowest average proportion of area protected (Table 4). Many of those estuaries are located near major urban centers, where the financial costs and logistical challenges associated with establishing and ensuring compliance of conservation designations are higher [45]. Protecting estuaries in highly populated areas will require stakeholder engagement, a component of EBM, to ensure that all relevant ecosystems and activities are considered, that priorities are established collectively, and that efforts are not duplicated [30], [46]. However, only the conservation of representative sites across the coast will ensure the continued viability of estuarine ecosystems and help Canada meet its international commitment to preserve 10% of each ecological region in the marine and coastal realm [46].

### Moderately Threatened Estuaries

Cluster 2 faced a moderate level of most threats (Table 3) and contained estuaries located primarily in the less populated ecosections of the south coast (Figure 3). These systems had the greatest impact from recreational fishing and forestry, suggesting that coordinating estuarine management with these two sectors might alleviate significant stresses. Regional planning is a means of engaging industries whose activities adversely affect an ecosystem from afar [47], such as the terrestrial activities like agriculture, forestry, and watershed development that affect estuaries in Clusters 2 and 3. Smith *et al.* [47] suggest that a system of local plans for highly used areas nested within regional plans might offer the most efficient solution. This suggestion meshes with recommendations from the Royal Society of Canada [48], who proposed that planning for DFO’s large ocean management areas should be tied to planning for the smaller coastal management areas because the areas are physically and biologically integrated.

### Minimally Threatened Estuaries

While some prioritize action in highly threatened areas, there are also calls to minimize conflict by proactively protecting areas required by few industries [49], [50]. Over 40% of the estuary-watershed systems assessed, represented by Cluster 1, were minimally affected (Table 3). This result is encouraging, suggesting that many of BC’s estuaries are relatively pristine. In comparison, research done for estuaries along the more heavily populated west coast of the United States found that only 16% were minimally affected [1]. The systems of Cluster 1 were located primarily in the lightly populated north coast ecosections (Figure 3). These sites faced a slightly greater average increase in future precipitation but because of the relatively low levels of other anthropogenic threats, the estuaries may be best poised to respond to the corresponding increase in water flow [51].

While the estuaries of Cluster 1 were the least affected by the combination of all threats, they did show moderate impacts from a few threats (Table 3). These estuaries are primarily located on the north coast, away from human settlements. However, small human populations do not guarantee minimal anthropogenic impacts [28] and some threats, such as forestry, shipping, and climate change, can influence even remote estuaries. Threats that impact all of the clusters are examples of regional issues that are likely managed most efficiently at a broad scale [11].

### Limitations in Data and Analysis

Cluster analysis is an inherently subjective statistical methodology relying on researcher judgment to gauge the integrity of the results. A strong theoretical foundation for the analysis helps exclude irrelevant variables that might distort results [41] and the extensive literature review of anthropogenic threats (Table 2) ensured only well-researched variables were incorporated. However, the lack of fine scale or historical spatial data on marine activities and climate change is a noted impediment to conservation and research efforts [17], [44].

Two specific data gaps should be noted. Observations of invasive species, one of the most important stresses in estuarine ecosystems [9], [19], have not been catalogued across the BC coast (D. Buffett, Ducks Unlimited Canada, personal communication). Instead, two vectors of invasive species, aquaculture and large vessel traffic [10], were used and the results can help identify highly disturbed estuaries most susceptible to invasion [8]. Perhaps the most important data gap is projected sea level rise. The shoreline armouring feature represents estuaries with barriers preventing adaptation to higher sea levels but does not consider spatial variations in elevation. The vulnerability of BC shorelines to sea level rise has been approximated by the BC Government using coarse elevation data and the physical parameters of shoreline segments [17]. However, better projections of the impact of SLR on estuaries require high resolution elevation data that are not currently available for much of the coast [17].

### Limitations in Implementation

Following the approach taken by Merrifield *et al.* [1], this analysis incorporated threats found throughout estuary-watershed systems. Estuaries link terrestrial watersheds with marine systems and the influences of the two realms both enhance [52] and threaten [3], [7] the ecosystem services provided by estuaries. However, while terrestrial, coastal, and marine environments are integrated, they are often not governed harmoniously. Considering the entire land to sea continuum is a more comprehensive approach to conservation and management advocated by both government agencies [23], [27] and researchers [7], [8], [34], [47], [53], despite the challenges associated with the large area and number of stakeholders involved. A failure to utilize EBM and explicitly consider threats originating across the land-sea continuum hinders effective estuary management and can undercut the success of protected areas [30]. Conservation efforts require the coordinated involvement of all relevant government bodies to facilitate successful integrated and adaptive planning [7], [21], [23], [34]. This will be particularly relevant for the estuaries in Clusters 2 and 3, which face a suite of significant marine and terrestrial threats. In BC, improved coordination may be underway as the BC government and First Nations are developing an EBM plan that considers the cumulative impacts of human activities (www.mappocean.org) and federal agencies are collaborating on a network of marine protected areas [46].

### Priorities for Management and Conservation

Land purchases such as those coordinated by the PECP play an integral role in the conservation of estuaries. To a certain extent, these purchases will likely always be done in an ad hoc manner, taking advantage of the generosity of landowners adjacent to estuaries. However, planning at a regional scale using biological and socioeconomic data can help ensure that management and conservation efforts are proactive, scientifically defensible, maximize available resources, and minimize impacts on local resource users [2], [25], [54]. A comprehensive network approach to protected areas can also help guarantee that protected areas fully realize their benefits [50]. To conserve estuarine ecosystem services, a network of protected areas must ensure that contributing sites include replications across the coast, maintain connectivity, and are adequately sized and protected [46].

Estuarine conservation and management would also benefit from further research into the cumulative impacts of estuarine threats. This analysis determined the specific threats facing BC’s estuaries but more information is required to understand how threats interact, in particular for the highly threatened estuaries in Clusters 2 and 3. Ban *et al.* [33] began this work for BC by extrapolating expert assessments from California on the vulnerability of marine habitats to different activities [35]. However, information on the level of impact of each threat is also required and is currently only available for a few of the regional scale datasets, a shortcoming identified in other jurisdictions [1]. The BCMCA has worked with industry representatives in the marine environment to validate the accuracy of spatial data [37] and continuing that work to generate a better representation of site usage and impact is an important next step.

Austin *et al.* [9] declared climate change to be the “foremost threat to biodiversity” in BC but a lack of spatial data (Table S3) make it challenging to incorporate the relevant threats. The BC estuaries identified as priorities for carbon sequestration [5] were shown to be highly threatened (Table 4). Protecting pristine carbon sequestration sites and restoring degraded areas through an EBM process has been referred to as “one of the strongest win-win mitigation efforts known today” [32] because safeguarding carbon sequestration requires maintaining estuarine structure and function, facilitating the continued provision of the other ecosystem services that estuaries provide. Emerging information on the capacity of estuaries to sequester carbon, along with better accounting methodologies, may bring renewed impetus to improve and fund coastal management from both the public and private sectors [22], [32], [42], [55].

## Conclusions

Estuaries are an important component of BC’s biodiversity and the ecosystem services they provide are well established. However, estuary degradation continues, in part due to a historical lack of integrated management and the investment required to develop and implement individual estuary management plans. Regional planning based on EBM that involves stakeholders across the entire land-sea continuum is required to prioritize sites for protection or restoration and ensure conservation benefits are realized.

By working at a broad scale and clustering estuaries based on their predominant threats, this analysis assesses human activities within BC’s estuaries scientifically and reveals that estuaries deemed most important for waterfowl habitat and carbon sequestration disproportionately face the highest threats. The results also highlight a north-south gradient of threats, with minimally threatened estuaries located predominantly on the north coast and highly threatened estuaries primarily on the south coast. This information can be combined with the PECP’s ecological ranking of estuaries to guide threat mitigation in marine, terrestrial, and coastal areas. Continuing to broaden our understanding of estuaries, including the cumulative impacts they face and the amount of carbon they sequester, will be essential to ensuring the sustained delivery of their vital ecosystems services.

## Supporting Information

Table S1 **Spatial datasets compiled to represent estuaries, watersheds, protected areas, and marine ecosections in British Columbia, Canada.**
(DOCX)

Table S2 **Spatial and non-spatial datasets compiled to represent estuarine threat variables in British Columbia, Canada.**
(DOCX)

Table S3 **Gaps in the spatial data available to represent estuarine threats identified in British Columbia, Canada.**
(DOCX)

## References

[1] Merrifield MS, Hines E, Liu X, Beck MW (2011) Building regional threat-based networks for estuaries in the Western United States. PLoS One 6(2): 1–10. 10.1371/journal.pone.0017407 PMC3046153 21387006

[2] Ryder JL, Kenyon JK, Buffett D, Moore K, Ceh M, et al.. (2007) An integrated biophysical assessment of estuarine habitats in British Columbia to assist regional conservation planning. Delta BC: Canadian Wildlife Service, Pacific and Yukon Region.

[3] Edgar GJ, Barrett NS, Braddon DJ, Last PR (2000) The conservation significance of estuaries: A classification of Tasmanian estuaries using ecological, physical and demographic attributes as a case study. Biological Conservation 92 383–397.

[4] Costanza R, d’Arge R, de Groot R, Farber S, Grasso M, et al (2007) The value of the world’s ecosystem services and natural capital. Nature 387 253–260.

[5] Campbell CR (2010) Blue carbon – British Columbia: The case for the conservation and enhancement of estuarine processes and sediments in BC. Victoria BC: Sierra Club of BC.

[6] Kelsey E (1995) The Pacific Estuary Conservation Program. Victoria BC: Pacific Estuary Conservation Program.

[7] Kennish MJ (2002) Environmental threats and environmental future of estuaries. Environmental Conservation 29(1): 78–107.

[8] Callaway GC, Zedler JB (2009) Conserving the diverse marshes of the Pacific coast. In Batzer DP, Baldwin AH, editors. Wetland habitat of North America: Ecology and conservation concerns. London: University of California Press. 285–307.

[9] Austin MA, Buffett DA, Nicolson DJ, Scudder GGE, Stevens V (2008) Taking nature’s pulse: The status of biodiversity in British Columbia. Victoria BC: Biodiversity BC.

[10] Environment Canada (1999) Draft Canada’s National Programme of Action (NPA) for the protection of the marine environment from land-based activities. Ottawa, ON: Environment Canada.

[11] Leschine TM, Ferriss BE, Bell KP, Bartz KK, MacWilliams S, et al (2003) Challenges and strategies for better use of scientific information in the management of coastal estuaries. Estuaries 26(4B): 1189–1204.

[12] Crossland CJ, Baird D, Ducrotoy JP, Lindebloom H (2005) The coastal zone – a domain of global interactions. In: Crossland, CJ, Kremer HH, Lindebloom HJ, Marshall Crossland JI, Le Tissier MDA, editors. Coastal fluxes in the anthropocene. New York: Springer-Verlag Berlin Heidelberg. 1–37.

[13] Scavia D, Field JC, Boesch DF, Buddemeier RW, Burkett V, et al (2002) Climate change impacts on U.S. coastal and marine ecosystems. Estuaries 25(2): 149–164.

[14] Galbraith H, Jones B, Park R, Clough J, Herrod-Julius S, et al (2002) Global climate change and sea level rise: Potential losses of intertidal habitat for shorebirds. Waterbirds 25(2): 173–183.

[15] Nicolas D, Chaalali A, Drouineau H, Lobry J, Uriarte A, et al (2011) Impact of global warming on European tidal estuaries: Some evidence of northward migration of estuarine fish species. Regional Environmental Change 11: 639–649.

[16] Feely RA, Alin SR, Newton J, Sabine CL, Warner M, et al (2010) The combined effects of ocean acidification, mixing, and respiration on pH and carbonate saturation in an urbanized estuary. Estuarine, Coastal and Shelf Science 88 442–449.

[17] Okey TA, Alidina HM, Lo V, Montenegro A, Jessen S (2012) Climate change impacts and vulnerabilities in Canada’s Pacific marine ecosystems. Vancouver BC: CPAWS BC and WWF-Canada.

[18] Ryder JL, Ceh M, Buffett D, Moore K (2006) Economic and conservation tenures in the intertidal areas of BC estuaries. In: Gilkeson L et al.., editors. Alive and inseparable: British Columbia’s coastal environment 2006. Victoria: Province of British Columbia. 35–41.

[19] Emmett R, Llansó R, Newton J, Thom R, Hornberger CM, et al (2000) Geographic signatures of North American West Coast estuaries. Estuaries 23(6): 765–792.

[20] DFO (2008) The role of the provincial and territorial governments in the oceans sector. Ottawa ON: Fisheries and Oceans Canada.

[21] Robb CK, Bodtker KM, Wright K, Lash J (2011) Commercial fisheries closures in marine protected areas on Canada’s Pacific coast: The exception, not the rule. Marine Policy 35: 309–316.

[22] Herr D, Pidgeon E, Laffoley D (2012) Blue carbon policy framework: Based on the discussion of the International Blue Carbon Policy Working Group. Gland Switzerland: International Union for Conservation of Nature.

[23] DFO (2002) Policy and operational framework for integrated management of estuarine, coastal and marine environments in Canada. Ottawa ON: Fisheries and Oceans Canada.

[24] Crowder L, Norse E (2008) Essential ecological insights for marine ecosystem-based management and marine spatial planning. Marine Policy 32: 772–778.

[25] Lotze HK, Lenihan HS, Bourque BJ, Bradbury RH, Cooke RG, et al (2006) Depletion, degradation and recovery potential of estuaries and coastal seas. Science 312: 1806–1809. 16794081 10.1126/science.1128035

[26] McLeod KL, Lubchenco J, Palumbi S, Rosenberg AA (2005) Scientific consensus statement on marine ecosystem-based management.

[27] DFO (2005) Canada’s oceans action plan: For present and future generations. Ottawa ON: Fisheries and Oceans Canada.

[28] Halpern BS, Walbridge S, Selkoe KA, Kappel CV, Micheli F, et al (2008) A global map of human impact on marine ecosystems. Science 319: 948–952. 18276889 10.1126/science.1149345

[29] Millennium Ecosystem Assessment (2005) Ecosystems and human well-being: A framework for assessment. Nairobi Kenya: United Nations Environment Programme.

[30] Agardy T, di Sciara GN, Christie P (2011) Mind the gap: Addressing the shortcomings of marine protected areas through large scale marine spatial planning. Marine Policy 35: 226–232.

[31] Douvere F (2008) The importance of marine spatial planning in advancing ecosystem-based sea use management. Marine Policy 32: 762–771.

[32] Nelleman C, Corcoran E, Duarte CM, Valdés L, DeYoung C, et al.. (2009) Blue carbon: A rapid response assessment. Arendal Norway: United Nations Environment Programme.

[33] Ban NC, Alidina HM, Ardron JA (2010) Cumulative impact mapping: Advances, relevance and limitations to marine management and conservation, using Canada’s Pacific waters as a case study. Marine Policy 34 876–886.

[34] Imperial MT, Hennessey TM (1996) The ecosystem-based approach to managing estuaries: An assessment of the National Estuary Program. Coastal Management 24 115–139.

[35] Teck SJ, Halpern BS, Kappel CV, Micheli F, Selkoe KA, et al (2010) Using expert judgment to estimate marine ecosystem vulnerability in the California Current. Ecological Applications 20(5): 1402–1416. 20666257 10.1890/09-1173.1

[36] Wang T, Hamann A, Spittlehouse DL, Murdock TQ (2012) ClimateWNA – high-resolution spatial climate data for western North America. Journal of Applied Meteorological and Climatology 51: 16–29.

[37] Ban NC, Bodtker KM, Nicolson D, Robb CK, Royle K, et al (2013) Setting the stage for marine spatial planning: Ecological and social data collation and analyses in Canada’s Pacific waters. Marine Policy 39 11–20.

[38] Google (2012) Google Earth, v. 6.1. Mountainview CA: Google, Inc.

[39] Quigley MP, Hall JA (1999) Recovery of macrobenthic communities after maintenance dredging in the Blyth Estuary, north-east England. Aquatic Conservation: Marine Freshwater Ecosystems 9: 63–73.

[40] Systat Software (2010) Systat, v 13. Chicago IL: Systat Software, Inc.

[41] Ketchen DJ Jr, Shook CL (1996) The application of cluster analysis in strategic management research: An analysis and critique. Strategic Management Journal 17: 441–458.

[42] Joppa LN, Pfaff A (2009) High and far: Biases in the location of protected areas. PLoS One 4(12): 1–6. 10.1371/journal.pone.0008273 PMC2788247 20011603

[43] Williams GL, Langer OE (2002) Review of estuary management plans in British Columbia. Vancouver BC: Fisheries and Oceans Canada.

[44] Gleason MG, Merrifield MS, Cook C, Davenport A, Shaw R (2006) Assessing gaps in marine conservation in California. Frontiers in Ecology and the Environment 4(5): 249–258.

[45] Balmford A, Gaston KJ, Blyth S, James A, Kapos V (2003) Global variation in terrestrial conservation costs, conservation benefits, and unmet conservation needs. Proc Natl Acad Sci U S A 100(3): 1046–1050. 12552123 10.1073/pnas.0236945100 PMC298723

[46] Government of Canada (2011) National framework for Canada’s network of marine protected areas. Ottawa ON: Fisheries and Oceans Canada.

[47] Smith HD, Maes F, Stojanovic TA, Ballinger RC (2011) The integration of land and marine spatial planning. Journal of Coastal Conservation 15: 291–303.

[48] Hutchings JA, Côté IM, Dodson JJ, Fleming IA, Jennings S, et al.. (2012) Sustaining Canadian marine biodiversity: Responding to the challenges posed by climate change, fisheries, and aquaculture. Ottawa ON: Royal Society of Canada.

[49] Brooks TM, Mittermeier RA, da Fonseca GAB, Gerlach J, Hoffmann M, et al (2006) Global biodiversity conservation priorities. Science 313(5783): 58–61. 16825561 10.1126/science.1127609

[50] Jessen S, Chan K, Côté I, Dearden P, De Santo E, et al. (2011) Science-based guidelines for MPAs and MPA networks in Canada. Vancouver BC: CPAWS.

[51] Baustian JJ, Mendelssohn IA, Hester MW (2012) Vegetation’s importance in regulating surface elevation in a coastal salt marsh facing elevated rates of sea level rise. Global Change Biology 18: 3377–3382.

[52] Barbier EB (2012) Progress and challenges in valuing coastal and marine ecosystem services. Review of Environmental Economics and Policy 6(1): 1–19.

[53] Sloan NA, Vance-Borland K, Ray GC (2007) Fallen between the cracks: Conservation linking land and sea. Conservation Biology 21(4): 897–898. 17650231 10.1111/j.1523-1739.2007.00731.x

[54] Klein CJ, Chan A, Kircher L, Cundiff AJ, Gardner N, et al (2008) Striking a balance between biodiversity conservation and socioeconomic viability in the design of marine protected areas. Conservation Biology 22(3): 691–700. 18325043 10.1111/j.1523-1739.2008.00896.x

[55] Luck GW, Chan KMA, Fay JP (2009) Protecting ecosystem services and biodiversity in the world’s watersheds. Conservation Letters 2: 179–188.

