# Supplementary material for: Assessing the Impact of Human Activities on British Columbia’s Estuaries
Source: PLoS One. 2014 Jun 17;9(6):e99578. doi: 10.1371/journal.pone.0099578 (PMC4061013; doi:10.1371/journal.pone.0099578)
Supplement: Table S1 — Spatial datasets compiled to represent estuaries, watersheds, protected areas, and marine ecosections in British Columbia, Canada. (DOCX) [file pone.0099578.s001.docx]

**Table S1.** Spatial datasets compiled to represent estuaries, watersheds, protected areas, and marine ecosections in British Columbia, Canada

| Feature | Custodian | Data Source | Date | Resolution |
| --- | --- | --- | --- | --- |
| 1. Estuaries | Pacific Estuary Conservation Program | <http://www.bcmca.ca> | 2007 | 1:10,000 |
| 2. Watersheds | Government of BC (Freshwater Atlas) | <http://www.data.gov.bc.ca/dbc/geo/index.page> | 2009 | 1:20,000 |
| 3. Protected Areas |  |  |  |  |
| Federal protected areas | Canadian Council on Ecological Areas | <http://www.ccea.org/en_carts.html> | 2012 | Variable |
| Provincial protected areas | Government of BC | <http://www.data.gov.bc.ca/dbc/geo/index.page> | 2012 | 1:20,000 |
| Private conservation areas | Ducks Unlimited Canada, The Nature Trust of BC, Environment Canada (Canadian Wildlife Service) | Contact Ducks Unlimited Canada for access to dataset | 2012 | Not provided |
| 4. Marine Ecosections | Government of BC (BC Marine Ecological Classification) | <http://www.data.gov.bc.ca/dbc/geo/index.page> | 2002 | 1:250,000 |

eral protected areas
