# Supplementary material for: Assessing the Impact of Human Activities on British Columbia’s Estuaries
Source: PLoS One. 2014 Jun 17;9(6):e99578. doi: 10.1371/journal.pone.0099578 (PMC4061013; doi:10.1371/journal.pone.0099578)
Supplement: Table S2 — Spatial and non-spatial datasets compiled to represent estuarine threat variables in British Columbia, Canada. (DOCX) [file pone.0099578.s002.docx]

**Table S2.** Spatial and non-spatial datasets compiled to represent estuarine threat variables in British Columbia, Canada

| Threats to Estuaries | Custodian | Data Source | Date | Resolution | Spatial Range of Influence (sq. m.) |
| --- | --- | --- | --- | --- | --- |
| 1. Development in watershed |  |  |  |  |  |
| Urban areas | Environment Canada (Canadian Wildlife Service) and Ducks Unlimited Canada | Contact Canadian Wildlife Service for access to dataset | 2000 | 25 m x 25 m | 30000 |
| Rail network | Natural Resources Canada (Atlas of Canada) | <http://www.geogratis.gc.ca> | 2008 | 1:1 million | 5000 |
| Routes (excluding ferry routes) | Government of BC | <http://www.data.gov.bc.ca/dbc/geo/index.page> | 2012 | Not provided | 5000 |
| 2. Agriculture in watershed |  |  |  |  |  |
| Agricultural areas | Environment Canada (Canadian Wildlife Service) and Ducks Unlimited Canada | Contact Canadian Wildlife Service for access to dataset | 2000 | 25 m x 25 m | 2000 |
| 3. Clearcutting in watershed |  |  |  |  |  |
| Provincial harvest depletion layer | Government of BC | <http://www.data.gov.bc.ca/dbc/geo/index.page> | 2012 | Not provided | 2000 |
| 4. Watershed pollution |  |  |  |  |  |
| Pulp and paper mills | Global Forest Watch Canada | <http://datawarehouse.globalforestwatch.ca/> | 2004 | Not provided | 30000 |
| Mines | Global Forest Watch Canada | <http://datawarehouse.globalforestwatch.ca/> | 2007 | Not provided | 30000 |
| Contamination closures | Environment Canada/Fisheries and Oceans Canada | <http://www.pac.dfo-mpo.gc.ca/fm-gp/contamination/biotox/index-eng.html> | 2012 | Not provided | Not applicable |
| 5. Freshwater diversions |  |  |  |  |  |
| Obstructions | Government of BC (Freshwater Atlas) | <http://www.data.gov.bc.ca/dbc/geo/index.page> | 2008 | 1:20,000 | Not applicable |
| 6. Shoreline armouring in estuary |  |  |  |  |  |
| Shorezone - manmade | Government of BC | <http://www.data.gov.bc.ca/dbc/geo/index.page> | 2011 | 1:20,000 | 0 |
| Dikes | Government of BC | <http://www.data.gov.bc.ca/dbc/geo/index.page> | 2004 | Not provided | Not applicable |
| 7. Development of estuary shoreline |  |  |  |  |  |
| Commercial recreational tenures | BC Marine Conservation Analysis | <http://www.bcmca.ca> | 2010 | 1:20,000 | 2000 |
| Log handling and storage | BC Marine Conservation Analysis | <http://www.bcmca.ca> | 2010 | 1:20,000 | 500 |
| Residential marine | BC Marine Conservation Analysis | <http://www.bcmca.ca> | 2010 | 1:20,000 | 2000 |
| Commercial and industrial (excluding commercial wharves) | BC Marine Conservation Analysis | <http://www.bcmca.ca> | 2010 | 1:20,000 | 2000 |
| Utilities | BC Marine Conservation Analysis | <http://www.bcmca.ca> | 2010 | 1:20,000 | 2000 |
| 8. Port facilities in estuary |  |  |  |  |  |
| Marinas and coastal facilities | BC Marine Conservation Analysis | <http://www.bcmca.ca> | 2010 | Not provided | 2000 |
| Anchorages and boat havens | BC Marine Conservation Analysis | <http://www.bcmca.ca> | 2010 | Not provided | 2000 |
| Ferry terminals | BC Marine Conservation Analysis | <http://www.bcmca.ca> | 2010 | 1:20,000 | 2000 |
| Tow boat reserves | BC Marine Conservation Analysis | <http://www.bcmca.ca> | 2010 | Not provided | 2000 |
| Commercial wharf tenures | BC Marine Conservation Analysis | <http://www.bcmca.ca> | 2010 | 1:20,000 | 2000 |
| Federal ports | Transport Canada/Port Authorities | Contact custodian for access to dataset | 2012 | Not provided | 2000 |
| 9. Dredging |  |  |  |  |  |
| Navigable Waters Works (dredging sites) | Transport Canada | Contact custodian for access to dataset | 2012 | Not provided | 500 |
| 10. Marine pollution |  |  |  |  |  |
| Vessel traffic (large vessels) | Oil in Canadian Waters Research Group | Contact custodian for access to dataset | 2010 | 500 m x 500 m | 30000 |
| Recreational boating routes | BC Marine Conservation Analysis | <http://www.bcmca.ca> | 2009 | Not provided | 2000 |
| 11. Aquaculture |  |  |  |  |  |
| Finfish aquaculture (marine) | Government of BC | <http://www.data.gov.bc.ca/dbc/geo/index.page> | 2012 | 1:20,000 | 2000 |
| Finfish aquaculture (marine) production | Fisheries and Oceans Canada | <http://www.pac.dfo-mpo.gc.ca/aquaculture/licence-permis/mar-eng.html> | 2012 | Non-spatial | Not applicable |
| Shellfish aquaculture | Government of BC | <http://www.data.gov.bc.ca/dbc/geo/index.page> | 2012 | 1:20,000 | 2000 |
| 12. Resource Extraction |  |  |  |  |  |
| Commercial geoduck fishery | Fisheries and Oceans Canada | <http://www.bcmca.ca> | 2000-2005 | 4 km x 4 km | Data too coarse for zone estimates |
| Commercial green urchin fishery | Fisheries and Oceans Canada | <http://www.bcmca.ca> | 2000-2005 | 4 km x 4 km |  |
| Commercial red urchin fishery | Fisheries and Oceans Canada | <http://www.bcmca.ca> | 2000-2005 | 4 km x 4 km |  |
| Commercial sea cucumber fishery | Fisheries and Oceans Canada | <http://www.bcmca.ca> | 2000-2005 | 4 km x 4 km |  |
| Commercial crab fishery | Fisheries and Oceans Canada | <http://www.bcmca.ca> | 2000-2005 | 4 km x 4 km |  |
| Commercial groundfish fishery | Fisheries and Oceans Canada | <http://www.bcmca.ca> | 1996-2004 | 4 km x 4 km |  |
| Commercial prawn fishery | Fisheries and Oceans Canada | <http://www.bcmca.ca> | 2001-2004 | 4 km x 4 km |  |
| Commercial schedule II fishery | Fisheries and Oceans Canada | <http://www.bcmca.ca> | 1996-2004 | 4 km x 4 km |  |
| Commercial shrimp trawl fishery | Fisheries and Oceans Canada | <http://www.bcmca.ca> | 1996-2004 | 4 km x 4 km |  |
| Commercial rockfish fishery | Fisheries and Oceans Canada | <http://www.bcmca.ca> | 1993-2004 | 4 km x 4 km |  |
| Commercial sardine fishery | Fisheries and Oceans Canada | <http://www.bcmca.ca> | 2001-2008 | 4 km x 4 km |  |
| Recreational groundfish fishery | BC Marine Conservation Analysis | <http://www.bcmca.ca> | 1993-2009 | Not provided | Data too coarse for zone estimates |
| Recreational anadromous fish fishery | BC Marine Conservation Analysis | <http://www.bcmca.ca> | 1993-2009 | Not provided |  |
| Recreational crab fishery | BC Marine Conservation Analysis | <http://www.bcmca.ca> | 1993-2009 | Not provided |  |
| Recreational prawn and shrimp fishery | BC Marine Conservation Analysis | <http://www.bcmca.ca> | 1993-2009 | Not provided |  |
| 13. Change in future precipitation |  |  |  |  |  |
| Projected precipitation change (1996-1990 to 2080) | University of British Columbia (ClimateBC_Map) | <http://www.genetics.forestry.ubc.ca/cfcg/ClimateBC40/Default.aspx> | 2012 | 0.0083 degrees | Not applicable |
