# Supplementary material for: Assessing the Impact of Human Activities on British Columbia’s Estuaries
Source: PLoS One. 2014 Jun 17;9(6):e99578. doi: 10.1371/journal.pone.0099578 (PMC4061013; doi:10.1371/journal.pone.0099578)
Supplement: Table S3 — Gaps in the spatial data available to represent estuarine threats identified in British Columbia, Canada. (DOCX) [file pone.0099578.s003.docx]

**Table S3.** Gaps in the spatial data available to represent estuarine threats identified in British Columbia, Canada

| Threats to Estuaries | Data Availability |
| --- | --- |
| Freshwater diversions |  |
| Run-of-river projects | Data were not available for existing sites. Transport Canada has data related to applications. Data may also be available from IPPwatch (http://www.IPPwatch.org). |
| Impaired waterways | Data do not exist. |
| Debris | Data do not exist. |
| Introduced species | Observation data are available from the Conservation Data Centre but comprehensive data for the coast do not exist. |
| Recreation |  |
| Low-impact tourism | Excluded because impact is considered minimal. |
| Port facilities in estuary |  |
| Cruise ship terminals | Comprehensive data do not exist. |
| Resource extraction |  |
| Commercial shrimp fisheries (humpback, pink, sidestripe) | Available data from Fisheries and Oceans Canada are too coarse (10 km x 10 km grid cells). |
| Sea surface temperature | Coastal data availability is limited. Remotely sensed data are affected by contamination due to the impact of the land signature. Data collected at lighthouses are not comprehensive for the coast. |
| Ocean acidification | Coastal data availability is limited. |
| Sea level rise | Manmade structures surrounding estuaries were used to highlight estuaries vulnerable to higher sea levels. A vulnerablity dataset is available from the Government of BC but the resolution is quite coarse. |
